# The Financial and Radiation Burden of Early Reimaging in Neurosurgical Patients: An Original Study and Review of the Literature

**DOI:** 10.7759/cureus.17383

**Published:** 2021-08-23

**Authors:** Rebecca Houston, Bandana Mahato, Tiffany Odell, Yasir R Khan, Deependra Mahato

**Affiliations:** 1 Neurosurgery, Desert Regional Medical Center, Palm Springs, USA

**Keywords:** neurosurgery, trauma, computed tomography, reimaging, ct

## Abstract

The computed tomographic (CT) scanner has become ubiquitous in healthcare. When trauma patients are imaged at facilities not equipped to care for them, imaging is often repeated at the receiving institution. CTs have clinical, financial, and resource costs, and eliminating unnecessary imaging will benefit patients, providers, and institutions. This paper reviews patterns of repetition of CT scans for transferred trauma patients and motivations underlying such behaviors via analysis of our Trauma Registry database and literature published in this area.

Neurosurgeons are fundamentally impactful in this decision-making process. The most commonly repeated scan is a CT head (CTH). More than ¼ of our patients receiving a clinically indicated repeat CTH also had a repeat scan of their cervical spine with no reason given for the cervical scan. Herein, we discuss our findings that both non-trauma center practitioners and non-neurosurgical staff at trauma centers cite a lower level of comfort with neuroradiology and fear of litigation as motivators in overzealous neuroimaging. As a result, inappropriate neurosurgical imaging is routinely ordered prior to transfer and again upon arrival at trauma centers. Education of non-neurosurgical staff is essential to prevent inappropriate neuroaxis imaging.

## Introduction

Computed tomographic (CT) scans are a critical component of care in the trauma setting, as they provide diagnostic information used for disposition and clinical management of acutely ill patients. CT scanners have become more common at facilities providing secondary care, such as urgent care centers and rural facilities. As a result, trauma patients have increasingly entered the medical system at a point with access to imaging, but without resources to provide adequate treatment of their degree of illness. Consequently, these patients are transferred to another institution for a higher level of care (HLOC).

This transition introduces an opportunity for duplication of imaging. There are clinical and opportunity (or resource utilization) costs associated with reimaging. Clinical costs include radiation exposure, intravenous contrast exposure, the potential for excessive spinal motion leading to spinal cord injury (SCI), and delay of definitive treatment. Resources extended for reimaging are financial, healthcare provider time, and decreased patient throughput with increased wait times for CT scans.

Our research evaluated the use of repeat CT (rCT) imaging at a tertiary hospital that is designated as a Level II trauma center. Our hospital receives transferred patients from multiple in-network (IN) and out-of-network (OON) non-trauma centers (NTCs) in Southern California. Our objectives were to describe CT usage in transferred trauma patients and to identify patient-level and system-level factors associated with rCT. We also examined the price of rCT and the radiation exposure associated with repeat scans. Furthermore, we sought to identify the rationale behind obtaining rCTs, and to determine what (if any) policies and procedures might be re-evaluated as a result. A review of the literature evaluated the scope of this issue. We summarize advances in technology and healthcare image sharing associated with a reduction in duplicate imaging.

## Materials and methods

This study was reviewed and approved by the Metrowest Medical Center Institutional Review Board with approval number 2018-178. The Desert Regional Medical Center (DRMC) Trauma Registry was reviewed for records of patients who were transferred from an outside facility to our hospital from January 2009 to May 2017. The term rCT was defined as a CT scan of the same anatomical region (with or without contrast) completed at the outside facility and again within 24 hours of arrival at DRMC. Dedicated scans of the thoracic or lumbar regions completed at DRMC were considered rCT when CT chest +/- abdomen and pelvis were completed at the transferring facility (which should have been used to create reconstructions).

Patient demographics, injury severity score (ISS), transferring facility, the reason given for rCT, and anatomical region of reimaging were obtained. Excluded from further analysis were patients under 18-years-old and those without rCT as defined above.

Data were grouped into reimaged vs. not reimaged. Reimaged was further stratified into IN vs. OON. Categorical variables were compared using Fisher exact test. Continuous variables were compared using Student’s t-test or the Mann-Whitney test. Statistical significance was set at p<0.05 with a CI of 95%. Statistical package for the social sciences (SPSS) Statistics for Windows, version 17.0 (SPSS Inc., Chicago, USA) was used for this analysis.

Radiation exposure was calculated by scan per body region using data available in the published literature regarding effective dose levels generated by scans for adult patients [1, 2]. Notably, there exists up to 32-fold variation in radiation doses reported for scans of the same body regions due to differences among machines and protocols. The costs per scan were assessed using the physician fee schedules from the Center of Medicare and Medicaid Services for Medicare Administrative Contractor (MAC) locality 0111262 Riverside, San Bernardino and Ontario, California.

We conducted a systematic review of the literature; bibliographic searches were conducted via electronic databases (Web of Science and PubMed) to access studies examining repeat CT scans and CT data-sharing technology.

## Results

Out of the 711 trauma patients transferred to our hospital, 368 (51.7%) had a duplicate CT completed. Independent predictors of rCT included ISS (median 10.9 vs. 9.3 for rCT vs. no repeat, respectively; p<0.05) and age (median 46 years old vs. 42 years old; p<0.05, Table 1).

**Table 1:** Bivariate Analysis of Factors Associated with Repeat Imaging

Variable (n=711)	Repeated (n=368)	Not Repeated (n=343)	P
Age, years (n=711)	45.8	42.2	<0.05
Injury Severity Score (n=711)	10.9	9.3	<0.05

Most commonly, no reason for repeat scan was recorded for each individual body region. For records citing a reason for a repeat scan, the most common reason given was a clinical need. The head was the most frequently scanned body region for this reason (75.8%, Table 2). Of the 126 patients who received rCT for a clinical reason and rCT cervical, 32 (26%) had no reason given for the cervical scan. Patients transferred with an unusable disc were more likely to be from an OON than IN facility (72% vs. 28%; p<0.05). The patients with duplicate scanning received a median of 9mSv of further radiation, with a median additional cost of $379. There was no statistically significant difference in radiation exposure or costs between IN and OON facilities. 

**Table 2:** Reasons for Repeating CT Scans rCT - Repeat Computed Tomography; CAP - Chest, Abdomen and Pelvis; OSH - Outside Hospital

rCT per Body Region Completed for Reason of: Clinical Indication				P Value
Body Region of rCT	In-Network	Out-of-Network	Total	
Head	49	42	91	
Maxillofacial	1	1	2	
Chest	2	4	6	
Abdomen & Pelvis	4	6	10	
Chest, Abdomen & Pelvis	3	2	5	
Lower Extremity	1	0	1	
Head & Cervical	2	3	5	
Total	62	58	120	.89
No Contrast Administered in Pre-transfer CT				
Body Region of rCT	In-Network	Out-of-Network	Total	
Chest	7	8	15	
Abdomen & Pelvis	7	12	19	
Pelvis	2	1	3	
CAP	9	8	17	
Neck	1	0	1	
Total	26	29	55	.69
Pre-transfer Images Not Available				
	In-Network	Out-of-Network	Total	
Images Not Received	4	2	6	
Disc Would Not Open	8	21	29	
Incomplete Images	6	0	6	
Total	18	23	41	.001
Reports Not Available				
	In-Network	Out-of-Network	Total	
	2	3	5	1
Dedicated Spinal Imaging Desired				
	In-Network	Out-of-Network	Total	
	5	10	15	.30
Poor Quality Imaging at OSH				
	In-Network	Out-of-Network	Total	
	3	4	7	1

## Discussion

Inappropriate medical imaging plagued the United States healthcare system in general [and in emergency departments (ED) in particular] for many years [3-5]. A 2009 report by the U.S. Department of Health and Human Services and a study by Kocher et al. documented the rise in prevalence of CT scanners with the concurrent observation that while ED visits increased nationwide by 30% from 1996 to 2007, CT scanning rose a disproportionate 330% [3, 4]. Subsequent efforts to curb the practice of inappropriate imaging came in the form of both physician initiatives, such as the Choosing Wisely campaign by the American Board of Internal Medicine Foundation with support from the American College of Physicians, as well as payor initiatives such as the Deficit Reduction Act of 2005 and Protecting Access to Medicare Act of 2014 [3, 5-9]. Studies demonstrate that while the rate of growth of usage of CT among older adults has slowed, the overall usage in this demographic has continued to increase despite these interventions [8].

**Figure 1:**
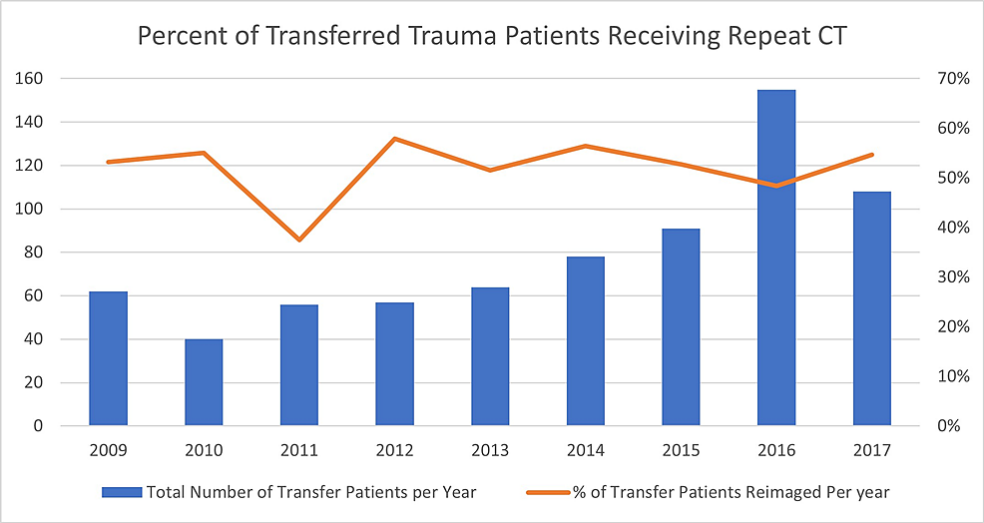
Percent of Transferred Trauma Patients Receiving Repeat CT

One previously identified subset of inappropriate imaging is the duplication of CT scanning in transferred trauma patients. CT is an indispensable aspect of emergency trauma and neurosurgical care. Imaging plays a critical role in the diagnosis and management of acutely ill patients. CT scanners have become increasingly available at facilities not equipped to provide a level of care correlated with the degree of injury in trauma patients. Examples of such non-trauma center facilities are urgent care and rural hospitals. A result of the increase in the prevalence of CT technology is that patients requiring neurosurgical and/or trauma care now frequently enter the medical system at a level of care with access to imaging, but without resources to provide adequate treatment of their acute traumatic and neurosurgical injuries. Consequently, these patients are transferred to another institution for a higher level of care. This transition introduces an opportunity for duplication of imaging, which is an internationally recognized problem [8, 10-12]. Overall rates of repeat CT scans in transferred trauma patients vary widely, from as low as 16% in a vertically integrated system to 90%; in our study, the value was found to be 52% (Figure 1; range 38%-56%).

CT scanning is an asset in trauma care as it is sensitive and minimally invasive. However, there are costs associated with duplication of CT scans which are threefold; clinical, financial, and those related to resource utilization.

Medically, CT scans carry risks associated with contrast exposure, the introduction of potentially excessive spinal motion in unstable spine injuries while moving the patient, delay of definitive treatment, and radiation exposure. Risks of contrast exposure include contrast-induced nephropathy, extravasation of contrast, associated sequelae such as compartment syndrome of the hand, and allergic reactions to contrast [13-17]. As the total volume of contrast is an independent risk factor for contrast-induced nephropathy, each independent exposure event should be carefully considered.

While various techniques are employed to prevent injury during bed transfers of patients with unstable spine injuries, no technique is entirely without risk. Up to 10% of patients with spinal cord injury (SCI) experience neurological deterioration after admission to the hospital [18]. In many studies, the cause of deterioration is listed as either unknown or not described. Cadaveric studies have demonstrated that common transfer techniques such as air-assisted lateral transfer devices, manual transfers, rolling boards, and sliding boards do cause substantial spinal motion, particularly angulation and translation [18-23]. The avoidance of inappropriate CT imaging, therefore, represents an opportunity to reduce the risk of iatrogenic SCI.

It is the recommendation of the Advanced Trauma Life Support course of the American College of Surgeons that imaging studies that may delay transfer to an HLOC should not be performed [24]. Multiple studies demonstrate improved outcomes in trauma patients with decreased time to a trauma center [11, 25-31]. A systematic review and meta-analysis by Celso et al. demonstrated that shorter transport times to hospitals that can provide definitive surgical care decreases mortality [29]. In 2011, Garwe et al. showed that transferred patients in their study had three times the two-week mortality compared to their counterparts who arrived directly at a trauma center (TC) [28]. Likewise, it’s also been well documented that CT scanning at NTC hospitals delays transfer [12, 16, 26, 30]. Nevertheless, times ranging from 67 minutes to 3 hours spent prior to transfer are shown in the literature [11, 12, 26, 27, 30]. In a 2018 study of one trauma network in Montreal, Bracco et al. found that sicker patients were spending more time in CT at a hospital unequipped to provide definitive treatment than were their arguably healthier counterparts [11].

Much debate has been made regarding the degree of health risks associated with exposure to radiation from medical imaging. The National Academy of Sciences’ Biologic Effects of Ionizing Radiation VII (BEIR VII) report has faced increasing criticism regarding the use of a linear, no-threshold (LNT) dose-response relationship between exposure to ionizing radiation and the development of radiation-induced solid cancers. A detailed discussion of the merits and limitations of BEIR VII is beyond the scope of this project. Still, a 2019 National Council on Radiation Protection review of the last decade of results found that many studies of solid cancer support the continued use of the LNT model in radiation protection [32-34]. As such, the exposure to ionizing radiation associated with CT scans should be minimized due to the potential for harmful effects, including the possibility of the development of radiation-induced malignancy [35].

Factors associated with repeat scans

Patient factors most commonly reported as associated with rCT are older age, higher ISS, and mechanism of injury (blunt rather than penetrating trauma) [5, 26, 30, 31, 36, 37]. Previous studies have consistently demonstrated older age to be a strong predictor of rCT [5, 30, 31, 36, 37]. Notably, a systematic review by Tung et al. showed that 3/6 studies meeting their definition of duplicate scans found an association with age in a bimodal distribution, with extremes of age more likely to have rCT [5]. We found that independent predictors of duplicate CT included ISS (median 10.9 vs. 9.3 for rCT vs. no-repeat, respectively; p<0.05) and age (median 46 years old vs. 42 years old; p<0.05). Our project otherwise excluded a review of patients under the age of 18. We did not find the male gender to be independently predictive of a repeat scan, a factor which has found mixed results in previous studies [5, 10]. Other factors in the literature noted to be associated with duplication of imaging are a good revised trauma score (RTS), and patients being transferred further [5, 10, 31]. Glasgow coma score (GCS) as an independent factor was mixed among studies; for many, a “lower GCS” was associated with an increased likelihood of repeat scan. However, there was no agreement on a cutoff value, and for multiple studies GCS was not found to be a statistically significant independent risk factor for repeat [5, 10, 26, 30, 38-40]. Factors that were not significant predictors were patient race and the status of Advanced Trauma Life Support (ATLS) certification of the ordering physician [5].

Systemic factors associated with the duplication of CT scans were broadly categorized as availability of referral center images to TC physicians and adequacy of those images to TC physicians.

Multiple studies found that a significant portion of repeat CT imaging was directly attributable to the lack of ability to review pre-transfer imaging; this was frequently cited as the most common reason for duplicate scans [5, 12, 13, 30, 41-43]. Failure to transfer data occurred when images were not sent with the patient, compact disc (CD) content was unviewable due to defective discs, non-digital imaging and communications in medicine (DICOM)-format CD’s were used, or other software problems leading to import failure or inability to the window, scroll or otherwise view images satisfactorily occurred. In our study, of the 368 total patients who received at least one rCT, patients received repeat scans for the reason that outside hospital (OSH) images were not available. At our institution, subcategories included images not received (15%), the CD would not open (71%), and incomplete images (15%) (Table 2). Patients transferred with an unusable disc were more likely to be from an OON facility than IN facility (72% vs. 28%; p<0.05). It was rare that scans were repeated solely for the reason OSH scan reports were not available (n=5) (Table 2). In comparison, one 2014 study reviewing transferred trauma patients with known spine injuries found that 41% of patients did not have referral center imaging (not limited to CTs) sent to the TC [43]. This is not a problem limited to the U.S., as a 2017 Swiss study found their most common reason for rCT was inadequate CT data transfer (39% of all rCTs), whereas no duplication occurred because of poor image quality [12].

Duplication of imaging produced at NTCs due to technical inadequacies such as lack of raw data required for spinal reconstructions and other inadequacies such as no intravenous (IV) contrast, or bone windows (despite scanning the desired body region) was common [5, 16, 30, 36, 42-44]. Despite only two cases in which discs could not be opened during their year-long study at a regional trauma center, Moore et al. found that greater than 33% of rCTs in their study were preventable, and were most commonly prompted by inadequate transfer of data from the referring center CT scan [44]. Specifically, they cited the lack of data required for reconstructions. This was the case in our data for only 15 total patients (4%) who received dedicated spinal imaging at our institution despite having received prior imaging of the chest, abdomen, and pelvis (Table 2). Moore also noted that the problem was limited to the compact disc-read only memory (CD-ROM) method transfer of data, as 100% of images transferred via picture archiving and communication system (PACS) included both the rendered images and the associated raw data used for reconstructions. The lack of reconstruction data was also responsible for 14% of rCTs in the previously cited study specific to trauma patients with spinal injuries [43]. Over 56% of rCTs performed at a Level I TC in 2012 serving a rural and metropolitan population were done because OSH scans were inadequate for workup due to shortcomings such as lack of contrast or lack of reconstructions [30]. Inadequate CT images were the most common reason behind 52% of rCTs, as noted by Flanagan et al. [36]. This was also identified as an opportunity for our center to decrease unnecessary imaging, with 55 total scans repeated due to lack of contrast (no statistical significance IN vs. OON) (Table 2).

Systemic factors associated with repetition of CTs, when technically adequate imaging was available, were perception of non-trauma center imaging quality and concerned radiologist's reads, physician preference, systematic bias, and environmental factors.

Referring hospitals have a reputation for poorer quality imaging than their TC counterparts. This reputation may not be entirely undeserved; a 2008 randomized survey of U.S. emergency departments found that smaller, rural and critical access hospitals had less access to higher-resolution CTs [45]. Indeed, sub-optimal quality imaging has repeatedly been named as a reason for duplication of imaging at TCs. The quality of the imaging has been described directly (e.g. imaging produced by one- to four-slice scanners providing less resolution, and technologist-dependent errors like contrast timing) and indirectly (via concern for missed injury despite the ability to view OSH imaging) [5, 10, 11, 15, 16, 30, 31, 36, 41, 42]. One study found that injuries not described by referring hospital scans which were found upon rCT of the same region upon arrival at TCs accounted for 54% of repeat imaging. These missed injuries were clinically significant 76% of the time and were attributed to poor quality of NTC imaging and NTC-radiologist inexperience [30]. However, other data suggest sub-optimal scans may still be viable. A retrospective review of records in one 2019 study found that the use of pre-transfer CT scans did not lead to any missed or miscategorized injuries [15]. Poor quality was rarely cited as the driving reason behind repeating a scan in our study at seven scans total (no statistical significance IN vs. OON) (Table 2).

The other factor associated with the use of NTC imaging, independent of the imaging itself, is NTC radiology reporting. Confidence in NTC radiology reports varies widely, with rates of discrepancies between NTC reports and TC radiologists' overreads of the same NTC images ranging from 0-49% [15, 25, 30, 43, 44, 46]. In our study, one patient had repeat imaging for reason given as NTC images were read by a nighthawk. One additional patient had one of his four scans repeated solely for disagreement between the NTC radiology report, which stated no acute fracture of the cervical spine, versus in-house radiologist interpretation of the same scan which did find injury. One level I trauma center did a retrospective study of 4.25 years of transferred adults with spine injuries and found that 86% of patients who had both the NTC imaging and report available also had a (non-required) TC radiologist report documented [43].

Given the apparent confidence bias, why not utilize more experienced TC radiologists (who arguably have increased pattern recognition of trauma imaging) to review NTC imaging? The answer is complex but may be succinctly summarized in saying that they won’t, and they can’t. Radiologist refusal and organizational policy prohibiting reads of outside imaging are preventative. Reasons include concern regarding a lack of protocol for billing repeat interpretation of images, policies that ban compensation for formal reads of OSH imaging, medicolegal issues, logistical challenges regarding transfer and storage of studies, and revenue loss [11, 36, 42, 43, 47-49]. Multiple compensation models have been studied in this arena. Flanagan et al. had the transferred imaging in their study read by in-house radiologists and charged a professional fee for the 10% of instances in which they found a discrepancy with the primary read [36]. A 2014 study from the Vanderbilt Orthopedic Institute noted that their hospital’s policy of compensating their radiologists for reading OSH imaging provides legal protection for TC physicians who do not receive official documentation of the OSH radiology report by the time the patient has arrived [43]. In a study dedicated to the matter, an American College of Surgeons Level I trauma center had in-house radiologists read outside images by way of the electronic medical record (EMR)-ordered consult. They addressed legal concerns by having NTC radiology reports uploaded to complete the consult [16]. The result was a statistically significant decrease in total CTs, including rCTs and those in every body region except the chest. Professional billing losses averaged $673 per patient, and there was an $800,000 annual decrease in hospital billing. The workload increased for radiologists without any increase in compensation, and concern was expressed that charging a fee for re-interpretation of the same images could be considered fraud. Though the consistent decrease in repeat scans is encouraging, challenges remain to widespread implementation of in-house reads of outside scans.

Imaging at these pre-transfer points of care is done for a variety of reasons, including the need to identify injuries, adequately triage patients, identify optimal transport methods, and document the need for referral to a higher level of care [15, 30]. Additional reasons for pre-transfer scanning at non-trauma centers cited in the literature include avoiding potential litigation, and the belief that the accepting facility expects imaging to be done [30]. While the diagnostic and management utility of CT scans for trauma patients is unquestionable, the common reasons cited for NTC scan, aforementioned studies showing significant delays to definitive care, and data suggesting that 36%-75% of CT scans performed prior to arrival at the TC will need to be repeated beg the question as to where in the trauma system scans should be performed [11].

Neurosurgical role in repeat imaging of transferred trauma patients

Neurosurgeons have a unique role to play in the effort to optimize rates of repeat scans. At our institution, the most commonly cited reason for repeat CT in transferred trauma patients was for clinical indications (Table 2). CTH was by far the most commonly repeated scan within this group and overall. This was consistent with the literature where CTH ranged from 32% to as much as 72% of all repeat CT scans [10, 12, 16, 27, 31, 47]. In our data, the number of repeat CTHs per patient within the first 24 hours of arrival ranged from one to three. This reflects the fact that serial imaging is indicated for various conditions associated with head trauma, such as intracranial bleeding and pneumocephalus, meeting certain criteria. However, there exist potential inefficiencies leading to unnecessary repeat CTH. Education of non-neurosurgical staff regarding indications and timing for repeat spinal and cranial imaging may reduce orders for scans that are ordered too soon after the OSH scan. A lower level of comfort with neuroradiology and fear of litigation also results in increased rates of neuroaxis scans, even in patients without head injury, loss of consciousness, amnesia, or neurological signs or symptoms [5].

Regarding head trauma, the paradigm that CTH at NTC will provide info to physicians at TC is questionable. Moreover, there exists data to suggest that even for patients with severe traumatic brain injury (TBI), CTH at referring institutions does not decrease time to neurosurgical intervention at the receiving trauma center [40]. On the contrary, a 2018 retrospective study at a level I trauma center in Iowa found the elapsed time between arrival at OSH and the start of the neurosurgical procedure for those who received OSH scan versus those who didn’t was an average of 5 hours, 6 minutes and 3 hours, 9 minutes, respectively [40]. One contributing factor was that more than 25% of those who were imaged at the OSH were reimaged at the trauma center.

An additional option for targeted improvement, we uniquely identified in our study, is the reflexive behavior to order scans of a neighboring region in patients who are already getting scanned for a clinical indication. This is particularly relevant to neurosurgery, as we found that 26% of patients who received both repeat CT head (rCTH) and rCT cervical with clinical indication listed as the reason for reimaging for CTH had no reason given for a repeat of the cervical scan. One possible contributing factor may be found in a 2014 study by Bible et al. which found that 72% of spine imaging is ordered by ED staff, not spine specialists [43].

Our institution demonstrated a successful pattern of utilizing reconstructions, with only 15 patients receiving dedicated spinal imaging over a nine-year period. Dedicated spinal imaging was defined as imaging of the thoracic and lumbar spine ordered at our hospital in instances in which the patient had a CT abdomen and pelvis +/- CT chest from which spinal imaging should have been available from reconstructions. Any subsequent thoracic or lumbar scans completed at our hospital were repetitive data collection, and while they were not outright repeat scans, they did incur unnecessary clinical, financial, and general resource burdens.

Proposed solutions

Inappropriate imaging of transferred trauma patients may be broadly classified as scans that should not have been completed and those for which the indication exists, but the timing of the scan is incorrect. In our analysis of the literature and our own data, we identified three categories of proposed changes to reduce repeat imaging in trauma patients. They are technology, education, and administration.

Technology allowing TC physicians and/or radiologists to reliably and efficiently evaluate NTC images may decrease the rate of unnecessary transfers [36]. Various means of image-sharing have been proposed. Integration of healthcare systems refers to a common owner or administrator; vertically integrated systems (VIS) have the same EMR and radiology information system (RIS), allowing access to patient data among disparate sites [50]. Integration of systems has long been studied as a means of improving care, while VIS has more recently been found to reach economies of scale and efficiencies of scale in excess of systems connected solely by affiliation and transfer agreements [14, 15, 36, 47, 50, 51]. A 2017 study of a Level I TC within a vertically integrated care system found the rate of repeat CT scanning to be significantly lower in patients transferred from an IN hospital (10.3%) compared to those transferred from an OON facility (41.1%) [51]. In their comparison of a university-based non-integrated system and a vertically integrated regional healthcare system, they found an overall repeat rate of 48% at the non-integrated system versus 16% at the VIS [51]. Beyond system-based integration, other proposals include a universal EMR, image transfer networks not limited by affiliations or hospital ownership, and internet-based virtual private networks (VPN) permitting direct connections between facilities [13, 25]. In fact, Banerjee et al. examined the results of the implementation of a cloud-based image sharing system in a regional trauma system and found that patients who had images imported using the new system had much lower rates of CT duplication than those who didn’t have their images imported (37% vs. 22%) [47].

Improvements in technology on a localized scale may also be effective. Difficulty in finding CDs has been expressed as a reason for duplication of images in the literature and our own data, particularly when multiple consult services are involved in a case [41]. A 2019 study at a Level I TC in Kansas updated/installed imaging software on all trauma bay computers to allow uploading of imaging as part of a larger effort to decrease the number of transfer scans that were unreadable due to incompatible hardware or software [15]. The result was a decrease in rCT’s done for a preventable reason from 25.8% to 14.3% of transferred patients.

Physician education may also decrease the duplication of scans. Physical examination findings of GCS<11, need airway intervention (including intubation), hypo or hypertension, and need for blood transfusion have been identified as factors necessitating transfer to a trauma center [30]. Standardization of an algorithm or system of indicators that is not exclusive to CT findings would decrease the number of unnecessary scans completed at NTCs. Educating providers regarding the most recent Advanced Trauma Life Support guidelines is recommended. Regarding neurosurgical scans at trauma centers, education of trauma system providers about expectations within an individual trauma system is critical, as evidenced by the ordering of unnecessary spinal axis scans and inappropriate timing of scans to assess intracranial bleed stability. Indeed, as much as 72% of spine imaging is completed in the ED and not ordered by a spine specialist [43]. Likewise, while CTH was the most commonly repeated study at our institution and throughout the literature, repeat scans are often ordered in the ED by non-neurosurgeons without regard to timing [10, 12, 16, 27, 31, 47]. Serial radiological exams play an important part in monitoring cranial injury; however, the propensity of non-neurosurgical staff to order repeat scans prematurely can contribute to an overall increased rate of CTH. Early neurosurgical consultation and education regarding the timing of repeat CTH may further reduce unnecessary imaging.

Changes in administrative policies also represent an opportunity to decrease repeat scans. As a result of a four-year study of trauma transfers to the University of North Carolina (UNC) and Duke University Hospitals (Duke), these institutions have tasked their trauma coordinators with following up with information technology for each individual instance in which data is unavailable due to inability to open the CD, or other software issues, with resultant improvement at one of the centers that implemented this change before the end of data collection [26]. As previously discussed, hospital policies may prevent in-house radiologist interpretation of transferred imaging. Quick et al. completed a study to evaluate a change in policy allowing for in-house interpretation (IHI) of OSH images; the result was a 56% reduction in rCTH, an 8% decrease in radiation exposure, and a $4,592 decrease in billing per patient [16]. Though challenges exist and the scope of implementation may be limited, for those systems in which radiologists, specialists, and hospital administrators may find an agreeable compromise, IHI of OSH images addresses the concern of NTC-radiologist inexperience as well as allowing for communication between TC physicians and the interpreting radiologist.

## Conclusions

Repeat CT scanning has clinical as well as financial costs and depletes resources. Systemically, improved image and report sharing through consortium agreements, improvements in technology, and education of providers at NTCs and TC’s alike represent opportunities to decrease the rate of unnecessary scanning. Policies allocating resources to follow-up on inefficiencies in image-sharing and allowing for in-hospital interpretation of outside imaging also help. A potential future area of study and subsequent intervention would be a more concise evaluation of decision-making at NTCs, with education regarding delays to HLOC for imaging that won’t change medical decision-making, as well as education regarding indications for contrasted versus non-contrasted images in trauma patients.

## References

[1] Dougeni E, Faulkner K, Panayiotakis G (2012). A review of patient dose and optimisation methods in adult and paediatric CT scanning. Eur J Radiol.

[2] Rehani MM (2015). Radiological protection in computed tomography and cone beam computed tomography. Ann ICRP.

[3] Larson DB, Johnson LW, Schnell BM, Salisbury SR, Forman HP (2011). National trends in CT use in the emergency department: 1995-2007. Radiology.

[4] Kocher KE, Meurer WJ, Fazel R, Scott PA, Krumholz HM, Nallamothu BK (2011). National trends in use of computed tomography in the emergency department. Ann Emerg Med.

[5] Tung M, Sharma R, Hinson JS, Nothelle S, Pannikottu J, Segal JB (2018). Factors associated with imaging overuse in the emergency department: a systematic review. Am J Emerg Med.

[6] (2021). S.1932 - Deficit Reduction Act of 2005. https://www.congress.gov/bill/109th-congress/senate-bill/1932.

[7] (2021). H.R.4302 - Protecting Access to Medicare Act of 2014. Medicare Act of.

[8] Smith-Bindman R, Kwan ML, Marlow EC (2019). Trends in use of medical imaging in US health care systems and in Ontario, Canada, 2000-2016. JAMA.

[9] (2021). Choosing Wisely® Promoting conversations between patients and clinicians. https://www.choosingwisely.org/..

[10] Blazak P, Hacking C, Presneill J, Reade M (2018). Early repeat computed tomographic imaging in transferred trauma and neurosurgical patients: incidence, indications and impact. J Med Imaging Radiat Oncol.

[11] Bracco D, Deckelbaum D, Artho G, Khwaja K, Mulder DS, Gruska J, Razek T (2018). Additional and repeated computed tomography in interfacility trauma transfers: room for standardization. Surgery.

[12] Hinzpeter R, Sprengel K, Wanner GA, Mildenberger P, Alkadhi H (2017). Repeated CT scans in trauma transfers: an analysis of indications, radiation dose exposure, and costs. Eur J Radiol.

[13] Sodickson A, Opraseuth J, Ledbetter S (2011). Outside imaging in emergency department transfer patients: CD import reduces rates of subsequent imaging utilization. Radiology.

[14] Watson JJ, Moren A, Diggs B (2016). A statewide teleradiology system reduces radiation exposure and charges in transferred trauma patients. Am J Surg.

[15] Vernon SA, Helmer SD, Ward JG, Haan JM (2019). Computed tomography in trauma patients accepted in transfer: missed injuries and rationale for repeat imaging. Can we do better?. Kans J Med.

[16] Quick JA, Bartels AN, Coughenour JP, Barnes SL (2013). Trauma transfers and definitive imaging: patient benefit but at what cost?. Am Surg.

[17] Belzunegui T, Louis CJ, Torrededia L, Oteiza J (2011). Extravasation of radiographic contrast material and compartment syndrome in the hand: a case report. Scand J Trauma Resusc Emerg Med.

[18] Hu CT, Dipaola CP, Conrad BP, Horodyski M, Del Rossi G, Rechtine GR (2013). Motion is reduced in the unstable spine with the use of mechanical devices for bed transfers. J Spinal Cord Med.

[19] Conrad BP, Rechtine G, Weight M, Clarke J, Horodyski M (2010). Motion in the unstable cervical spine during hospital bed transfers. J Trauma.

[20] DiPaola CP, DiPaola MJ, Conrad BP, Horodyski M, Del Rossi G, Sawers A, Rechtine GR 2nd (2008). Comparison of thoracolumbar motion produced by manual and Jackson-table-turning methods. Study of a cadaveric instability model. J Bone Joint Surg Am.

[21] Horodyski M, Weight M, Conrad B, Bearden B, Kimball J, Rechtine G (2009). Motion generated in the unstable lumbar spine during hospital bed transfers. J Spinal Disord Tech.

[22] Horodyski M, Conrad BP, Del Rossi G, DiPaola CP, Rechtine GR 2nd (2011). Removing a patient from the spine board: is the lift and slide safer than the log roll?. J Trauma.

[23] Rechtine GR, Del Rossi G, Conrad BP, Horodyski M (2004). Motion generated in the unstable spine during hospital bed transfers. J Trauma.

[24] (2021). ATLS offers new insights into managing trauma patients. https://bulletin.facs.org/2018/06/atls-10th-edition-offers-new-insights-into-managing-trauma-patients/..

[25] Haley T, Ghaemmaghami V, Loftus T, Gerkin RD, Sterrett R, Ferrara JJ (2009). Trauma: the impact of repeat imaging. Am J Surg.

[26] Emick DM, Carey TS, Charles AG, Shapiro ML (2012). Repeat imaging in trauma transfers: a retrospective analysis of computed tomography scans repeated upon arrival to a Level I trauma center. J Trauma Acute Care Surg.

[27] Berkseth TJ, Mathiason MA, Jafari ME, Cogbill TH, Patel NY (2014). Consequences of increased use of computed tomography imaging for trauma patients in rural referring hospitals prior to transfer to a regional trauma centre. Injury.

[28] Garwe T, Cowan LD, Neas BR, Sacra JC, Albrecht RM (2011). Directness of transport of major trauma patients to a level I trauma center: a propensity-adjusted survival analysis of the impact on short-term mortality. J Trauma.

[29] Celso B, Tepas J, Langland-Orban B, Pracht E, Papa L, Lottenberg L, Flint L (2006). A systematic review and meta-analysis comparing outcome of severely injured patients treated in trauma centers following the establishment of trauma systems. J Trauma.

[30] Bonds MM, Hersperger S, Garwe T (2017). Adequacy and accuracy of nontrauma center computed tomography: What are we missing?. J Trauma Acute Care Surg.

[31] Wan Y, Stewart KE, Lansdale MQ (2018). Repeat computed tomography scans among inter-facility transferred major trauma patients in Oklahoma, 2009-2015. Emerg Radiol.

[32] Shore RE, Beck HL, Boice JD Jr (2019). Recent epidemiologic studies and the linear no-threshold model for radiation protection-considerations regarding NCRP commentary 27. Health Phys.

[33] Boice JD Jr (2017). The linear nonthreshold (LNT) model as used in radiation protection: an NCRP update. Int J Radiat Biol.

[34] National Research Council (2006). Health Risks from Exposure to Low Levels of Ionizing Radiation.

[35] Cook SH, Fielding JR, Phillips JD (2010). Repeat abdominal computed tomography scans after pediatric blunt abdominal trauma: missed injuries, extra costs, and unnecessary radiation exposure. J Pediatr Surg.

[36] Flanagan PT, Relyea-Chew A, Gross JA, Gunn ML (2012). Using the Internet for image transfer in a regional trauma network: effect on CT repeat rate, cost, and radiation exposure. J Am Coll Radiol.

[37] Young AJ, Meyers KS, Wolfe L, Duane TM (2012). Repeat computed tomography for trauma patients undergoing transfer to a Level I trauma center. Am Surg.

[38] Fabbri A, Servadei F, Marchesini G, Stein SC, Vandelli A (2008). Observational approach to subjects with mild-to-moderate head injury and initial non-neurosurgical lesions. J Neurol Neurosurg Psychiatry.

[39] Farach SM, Danielson PD, Amankwah EK, Chandler NM (2015). Repeat computed tomography scans after pediatric trauma: results of an institutional effort to minimize radiation exposure. Pediatr Surg Int.

[40] Tonui PM, Spilman SK, Pelaez CA, Mankins MR, Sidwell RA (2018). Head CT before transfer does not decrease time to craniotomy for TBI patients. Am Surg.

[41] Lu MT, Tellis WM, Fidelman N, Qayyum A, Avrin DE (2012). Reducing the rate of repeat imaging: import of outside images to PACS. AJR Am J Roentgenol.

[42] Gupta R, Greer SE, Martin ED (2010). Inefficiencies in a rural trauma system: the burden of repeat imaging in interfacility transfers. J Trauma.

[43] Bible JE, Kadakia RJ, Kay HF, Zhang CE, Casimir GE, Devin CJ (2014). Repeat spine imaging in transferred emergency department patients. Spine (Phila Pa 1976).

[44] Moore HB, Loomis SB, Destigter KK (2013). Airway, breathing, computed tomographic scanning: duplicate computed tomographic imaging after transfer to trauma center. J Trauma Acute Care Surg.

[45] Ginde AA, Foianini A, Renner DM, Valley M, Camargo CA Jr (2008). Availability and quality of computed tomography and magnetic resonance imaging equipment in U.S. emergency departments. Acad Emerg Med.

[46] Sung JC, Sodickson A, Ledbetter S (2009). Outside CT imaging among emergency department transfer patients. J Am Coll Radiol.

[47] Banerjee A, Zosa BM, Allen D, Wilczewski PA, Ferguson R, Claridge JA (2016). Implementation of an image sharing system significantly reduced repeat computed tomographic imaging in a regional trauma system. J Trauma Acute Care Surg.

[48] Robinson JD, McNeeley MF (2012). Transfer patient imaging: a survey of members of the American Society of Emergency Radiology. Emerg Radiol.

[49] Mendelson DS, Bak PR, Menschik E, Siegel E (2008). Informatics in radiology: image exchange: IHE and the evolution of image sharing. Radiographics.

[50] Liepert AE, Bledsoe J, Stevens MH, Cochran A (2014). Protecting trauma patients from duplicated computed tomography scans: the relevance of integrated care systems. Am J Surg.

[51] Bledsoe J, Liepert AE, Allen TL (2017). The salutary effect of an integrated system on the rate of repeat CT scanning in transferred trauma patients: Improved costs and efficiencies. Am J Surg.

